# Supplementary material for: A Humanized Yeast Phenomic Model of Deoxycytidine Kinase to Predict Genetic Buffering of Nucleoside Analog Cytotoxicity
Source: Genes (Basel). 2019 Sep 30;10(10):770. doi: 10.3390/genes10100770 (PMC6826991; doi:10.3390/genes10100770)
Supplement: Supplementary file 1 [file genes-10-00770-s001.zip › Additional_Files/Additional_File7_GOTermHeatmaps.zip!/Additional_File7_GOTermHeatmaps/protein-DNA_complex_assembly.pdf]

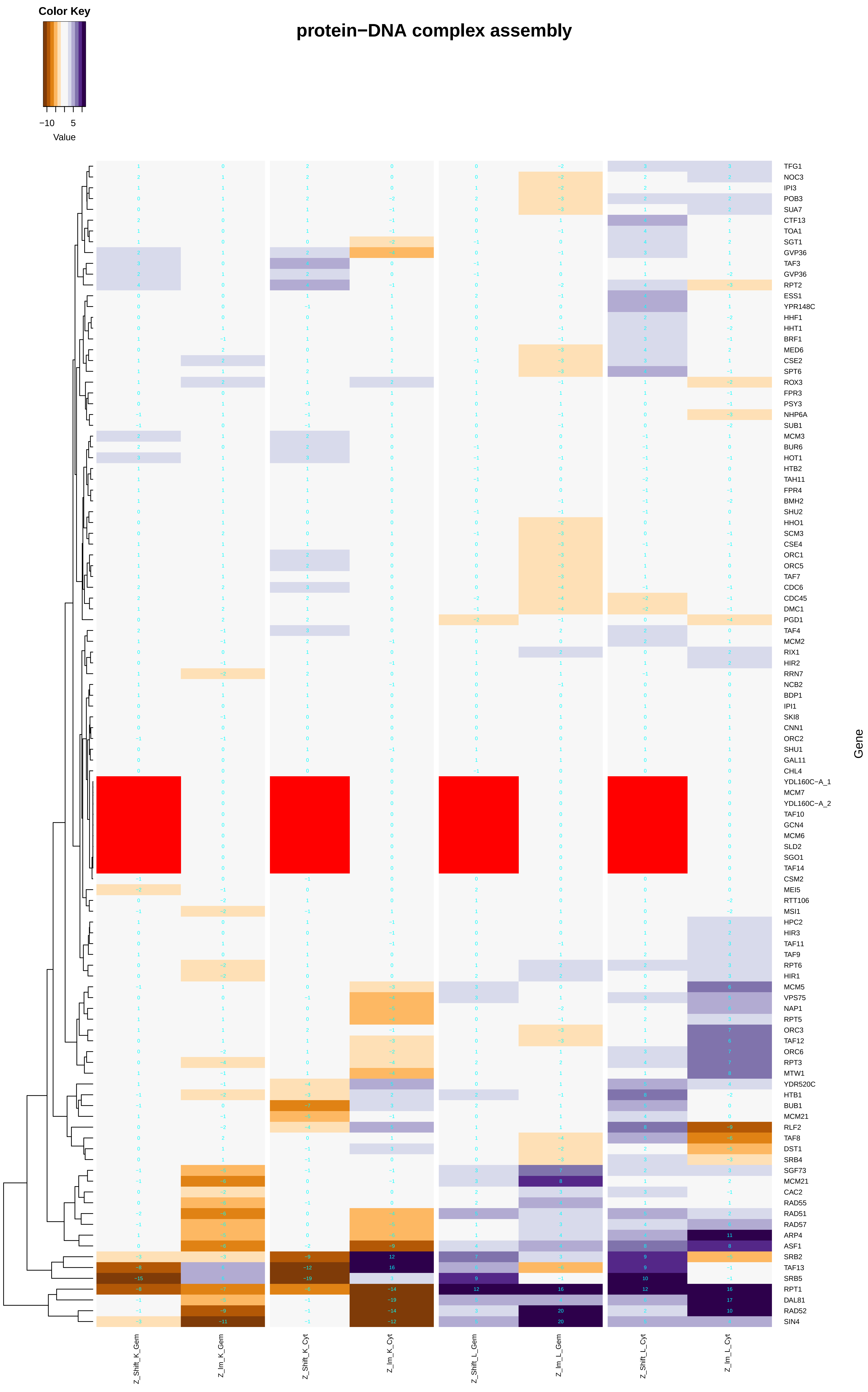

Color Key
−10
5
Value
protein−DNA complex assembly
TFG1
NOC3
IPI3
POB3
SUA7
CTF13
TOA1
SGT1
GVP36
TAF3
GVP36
RPT2
ESS1
YPR148C
HHF1
HHT1
BRF1
MED6
CSE2
SPT6
ROX3
FPR3
PSY3
NHP6A
SUB1
MCM3
BUR6
HOT1
HTB2
TAH11
FPR4
BMH2
SHU2
HHO1
SCM3
CSE4
ORC1
ORC5
TAF7
CDC6
CDC45
DMC1
PGD1
TAF4
MCM2
RIX1
HIR2
RRN7
NCB2
BDP1
IPI1
SKI8
CNN1
ORC2
SHU1
GAL11
CHL4
YDL160C−A_1
MCM7
YDL160C−A_2
TAF10
GCN4
MCM6
SLD2
SGO1
TAF14
CSM2
MEI5
RTT106
MSI1
HPC2
HIR3
TAF11
TAF9
RPT6
HIR1
MCM5
VPS75
NAP1
RPT5
ORC3
TAF12
ORC6
RPT3
MTW1
YDR520C
HTB1
BUB1
MCM21
RLF2
TAF8
DST1
SRB4
SGF73
MCM21
CAC2
RAD55
RAD51
RAD57
ARP4
ASF1
SRB2
TAF13
SRB5
RPT1
DAL81
RAD52
SIN4
Gene
Z_Shift_K_Gem
Z_Im_K_Gem
Z_Shift_K_Cyt
Z_Im_K_Cyt
Z_Shift_L_Gem
Z_Im_L_Gem
Z_Shift_L_Cyt
Z_Im_L_Cyt

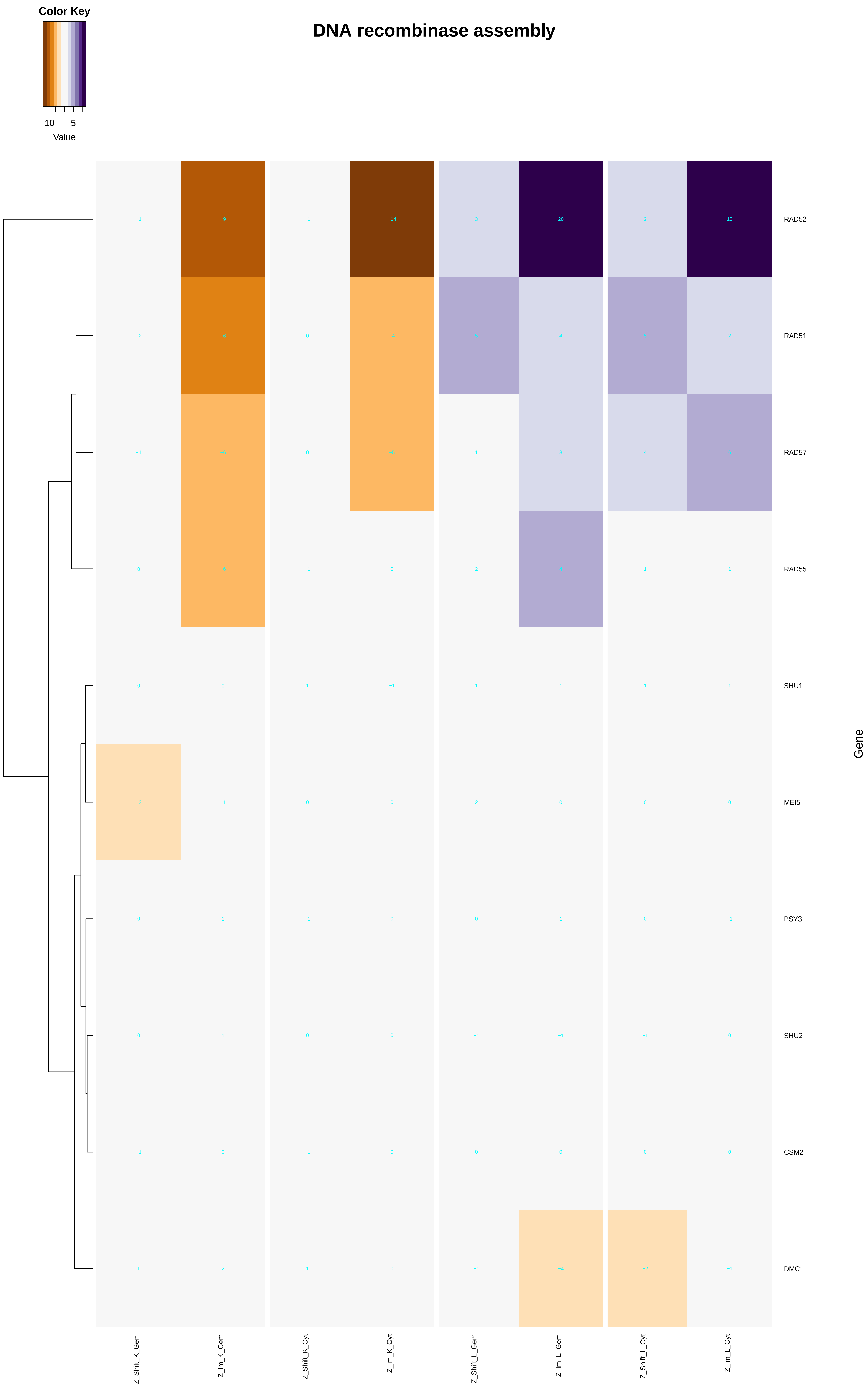
DNA recombinase assembly
Color Key
−10
5
Value
RAD52
RAD51
RAD57
RAD55
SHU1
MEI5
PSY3
SHU2
CSM2
DMC1
Gene
Z_Shift_K_Gem
Z_Im_K_Gem
Z_Shift_K_Cyt
Z_Im_K_Cyt
Z_Shift_L_Gem
Z_Im_L_Gem
Z_Shift_L_Cyt
Z_Im_L_Cyt

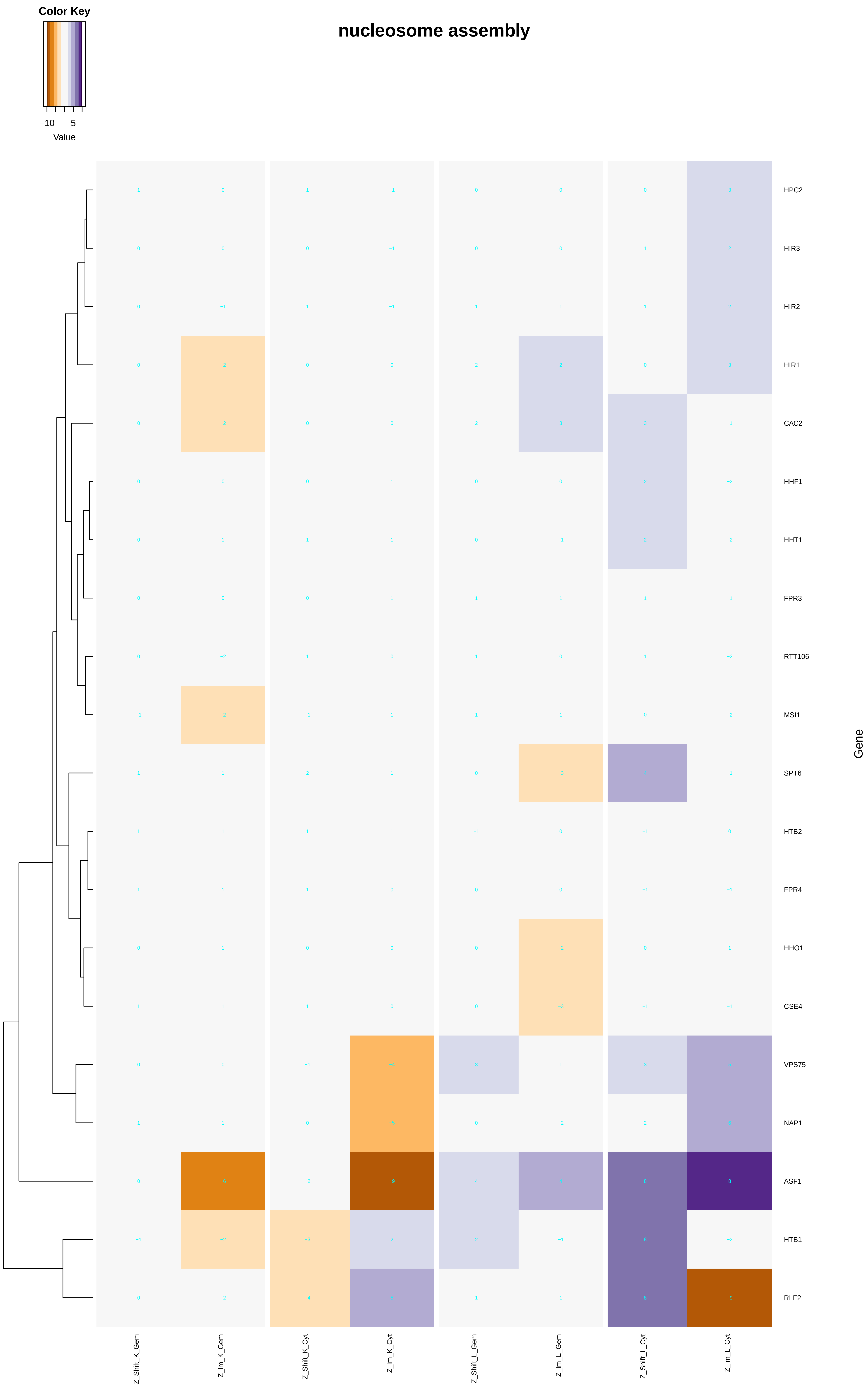

Color Key
−10
5
Value
nucleosome assembly
HPC2
HIR3
HIR2
HIR1
CAC2
HHF1
HHT1
FPR3
RTT106
MSI1
SPT6
HTB2
FPR4
HHO1
CSE4
VPS75
NAP1
ASF1
HTB1
RLF2
Gene
Z_Shift_K_Gem
Z_Im_K_Gem
Z_Shift_K_Cyt
Z_Im_K_Cyt
Z_Shift_L_Gem
Z_Im_L_Gem
Z_Shift_L_Cyt
Z_Im_L_Cyt

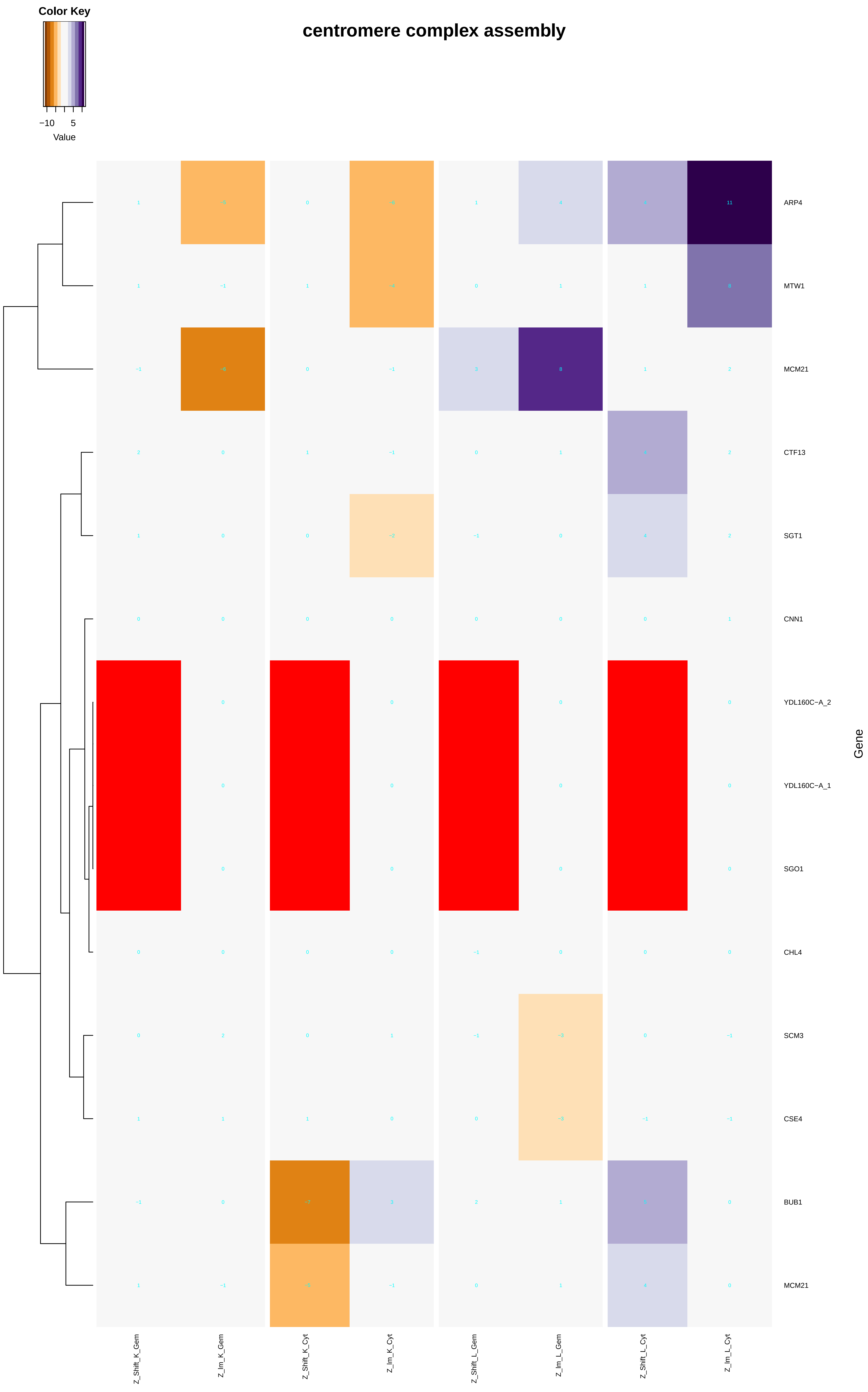

Color Key
−10
5
Value
centromere complex assembly
ARP4
MTW1
MCM21
CTF13
SGT1
CNN1
YDL160C−A_2
YDL160C−A_1
SGO1
CHL4
SCM3
CSE4
BUB1
MCM21
Gene
Z_Shift_K_Gem
Z_Im_K_Gem
Z_Shift_K_Cyt
Z_Im_K_Cyt
Z_Shift_L_Gem
Z_Im_L_Gem
Z_Shift_L_Cyt
Z_Im_L_Cyt

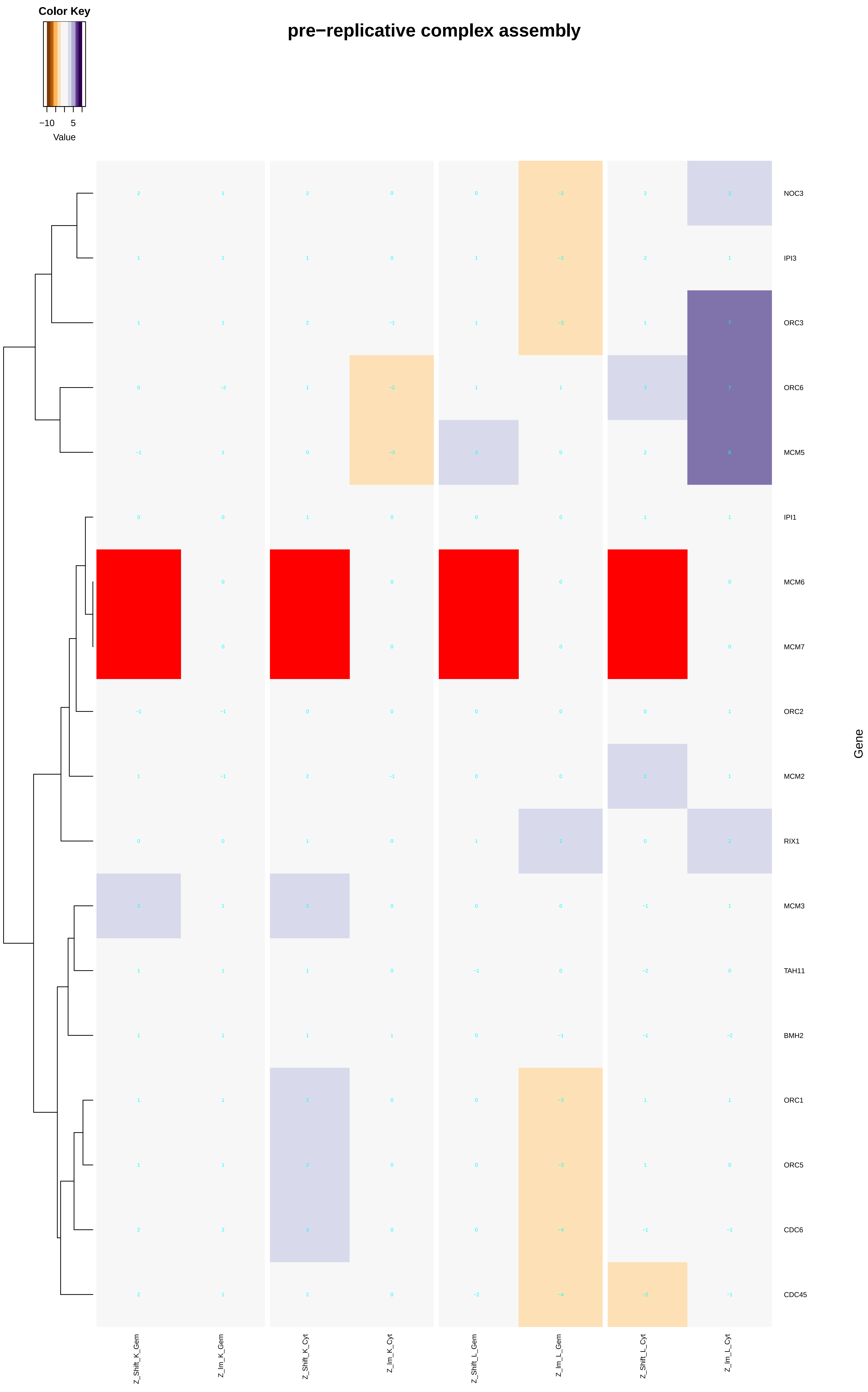

Color Key
−10
5
Value
pre−replicative complex assembly
NOC3
IPI3
ORC3
ORC6
MCM5
IPI1
MCM6
MCM7
ORC2
MCM2
RIX1
MCM3
TAH11
BMH2
ORC1
ORC5
CDC6
CDC45
Gene
Z_Shift_K_Gem
Z_Im_K_Gem
Z_Shift_K_Cyt
Z_Im_K_Cyt
Z_Shift_L_Gem
Z_Im_L_Gem
Z_Shift_L_Cyt
Z_Im_L_Cyt

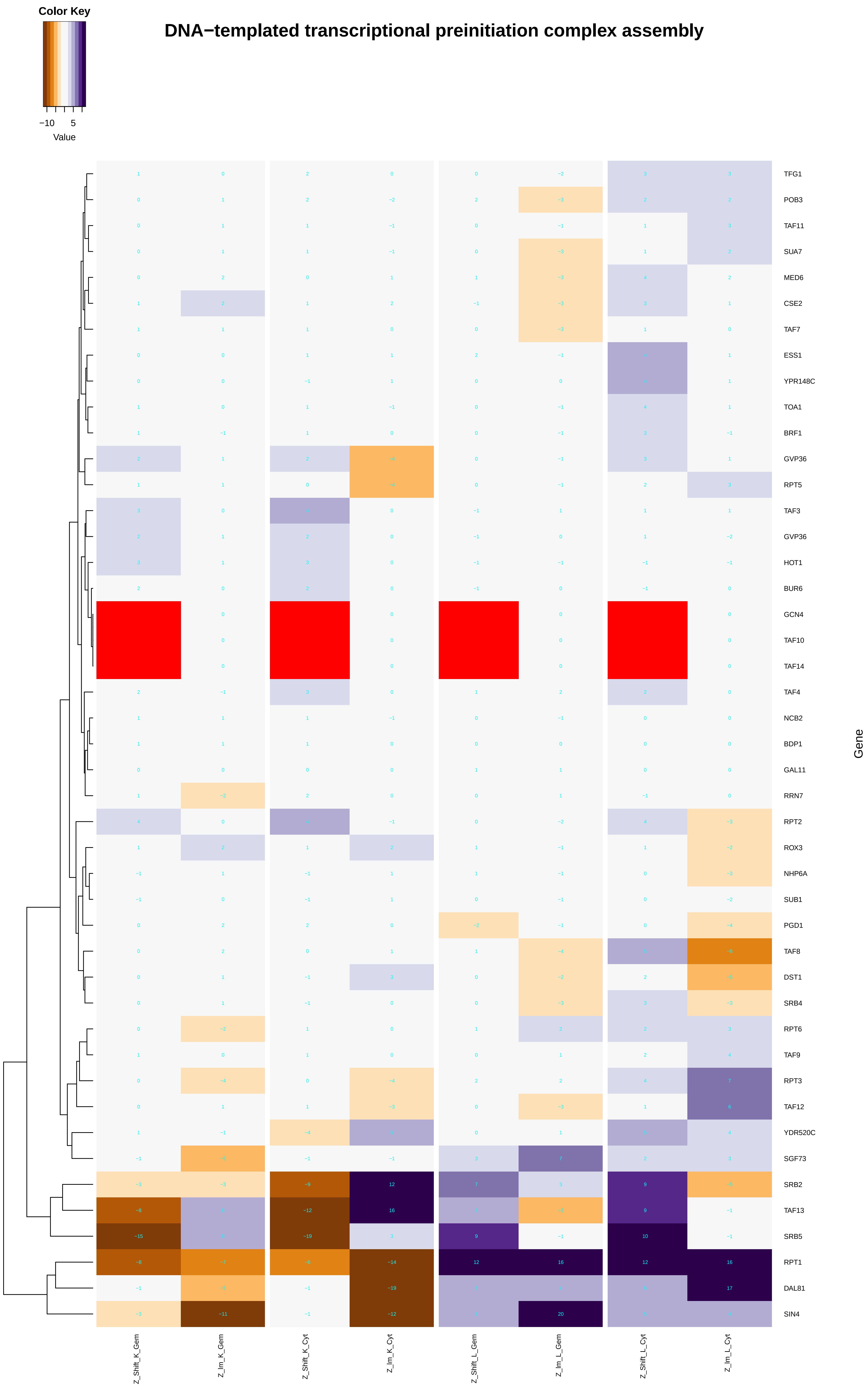

Color Key
−10
5
Value
DNA−templated transcriptional preinitiation complex assembly
TFG1
POB3
TAF11
SUA7
MED6
CSE2
TAF7
ESS1
YPR148C
TOA1
BRF1
GVP36
RPT5
TAF3
GVP36
HOT1
BUR6
GCN4
TAF10
TAF14
TAF4
NCB2
BDP1
GAL11
RRN7
RPT2
ROX3
NHP6A
SUB1
PGD1
TAF8
DST1
SRB4
RPT6
TAF9
RPT3
TAF12
YDR520C
SGF73
SRB2
TAF13
SRB5
RPT1
DAL81
SIN4
Gene
Z_Shift_K_Gem
Z_Im_K_Gem
Z_Shift_K_Cyt
Z_Im_K_Cyt
Z_Shift_L_Gem
Z_Im_L_Gem
Z_Shift_L_Cyt
Z_Im_L_Cyt

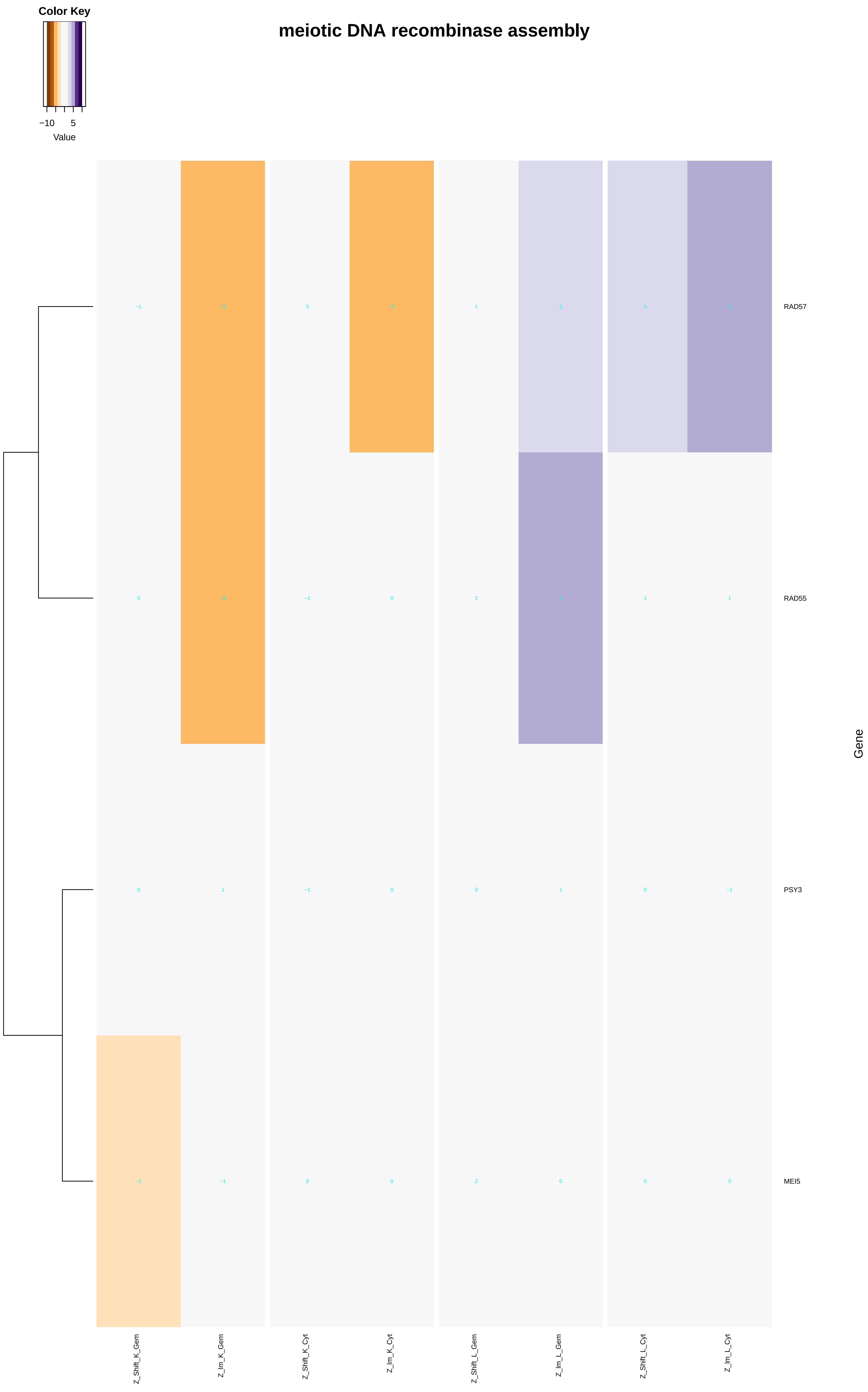
meiotic DNA recombinase assembly
Color Key
−10
5
Value
RAD57
RAD55
PSY3
MEI5
Gene
Z_Shift_K_Gem
Z_Im_K_Gem
Z_Shift_K_Cyt
Z_Im_K_Cyt
Z_Shift_L_Gem
Z_Im_L_Gem
Z_Shift_L_Cyt
Z_Im_L_Cyt

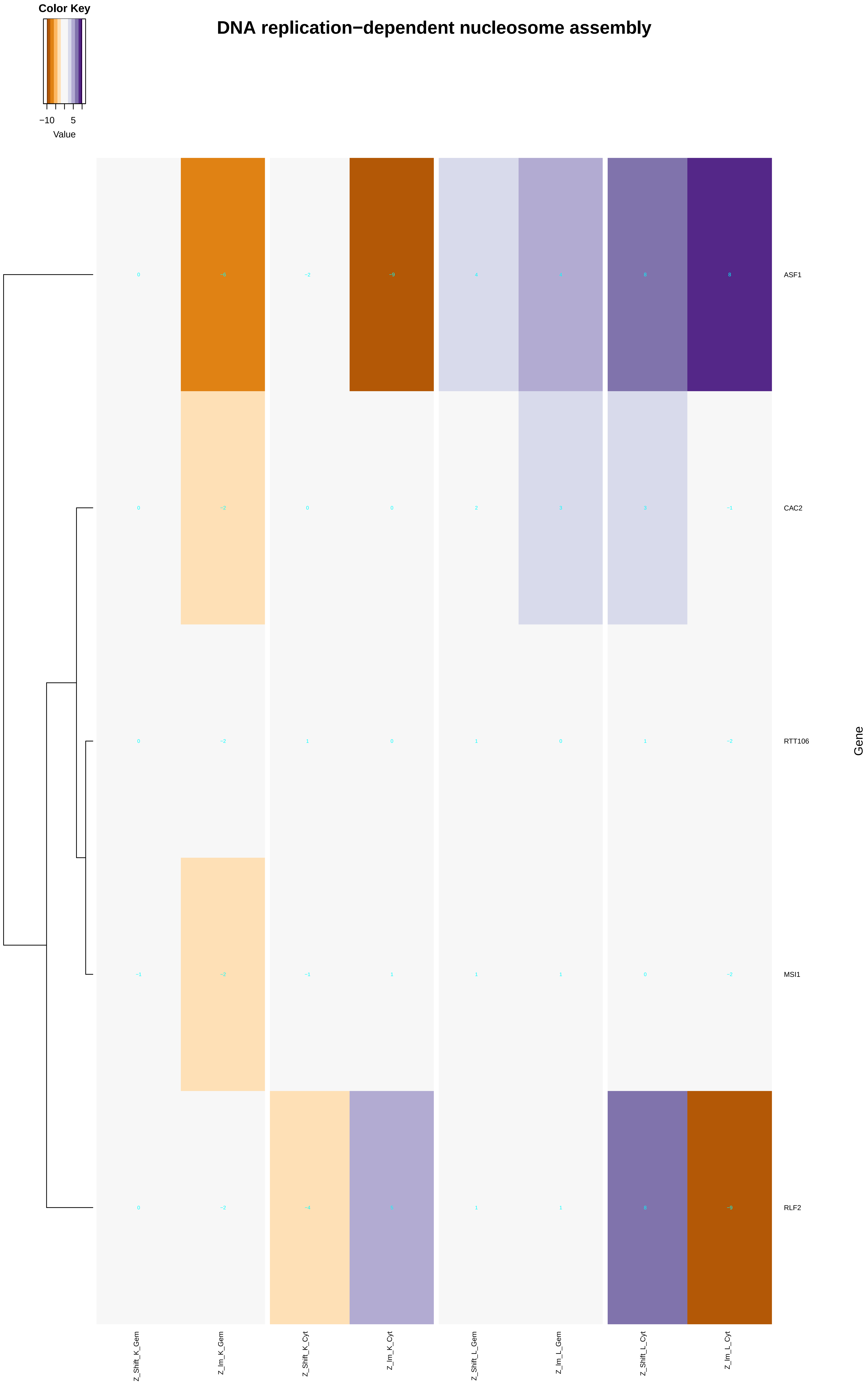

Color Key
−10
5
Value
DNA replication−dependent nucleosome assembly
ASF1
CAC2
RTT106
MSI1
RLF2
Gene
Z_Shift_K_Gem
Z_Im_K_Gem
Z_Shift_K_Cyt
Z_Im_K_Cyt
Z_Shift_L_Gem
Z_Im_L_Gem
Z_Shift_L_Cyt
Z_Im_L_Cyt

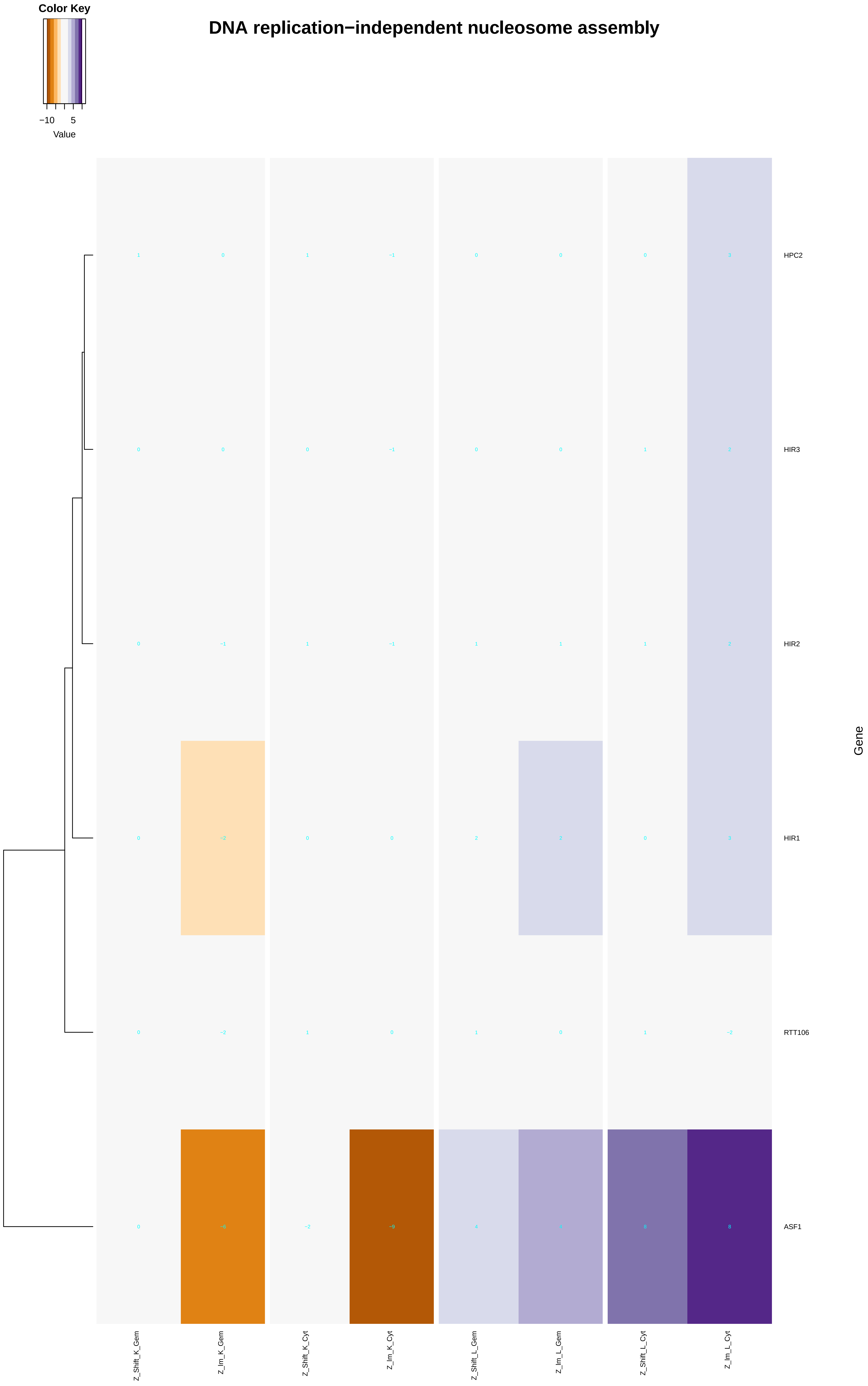

Color Key
−10
5
Value
DNA replication−independent nucleosome assembly
HPC2
HIR3
HIR2
HIR1
RTT106
ASF1
Gene
Z_Shift_K_Gem
Z_Im_K_Gem
Z_Shift_K_Cyt
Z_Im_K_Cyt
Z_Shift_L_Gem
Z_Im_L_Gem
Z_Shift_L_Cyt
Z_Im_L_Cyt

pre−replicative complex assembly involved in cell cycle DNA replication

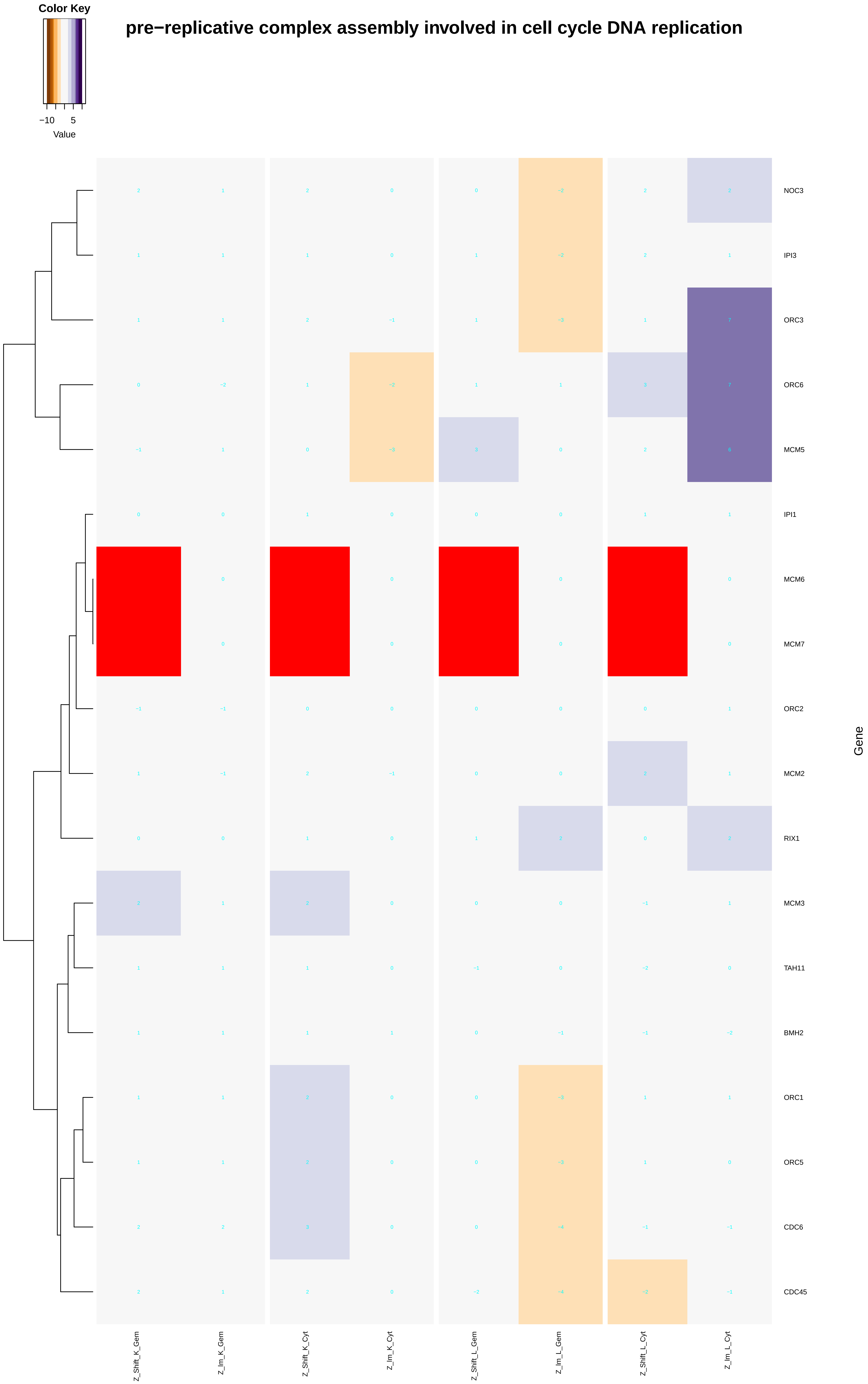

RNA polymerase II transcriptional preinitiation complex assembly

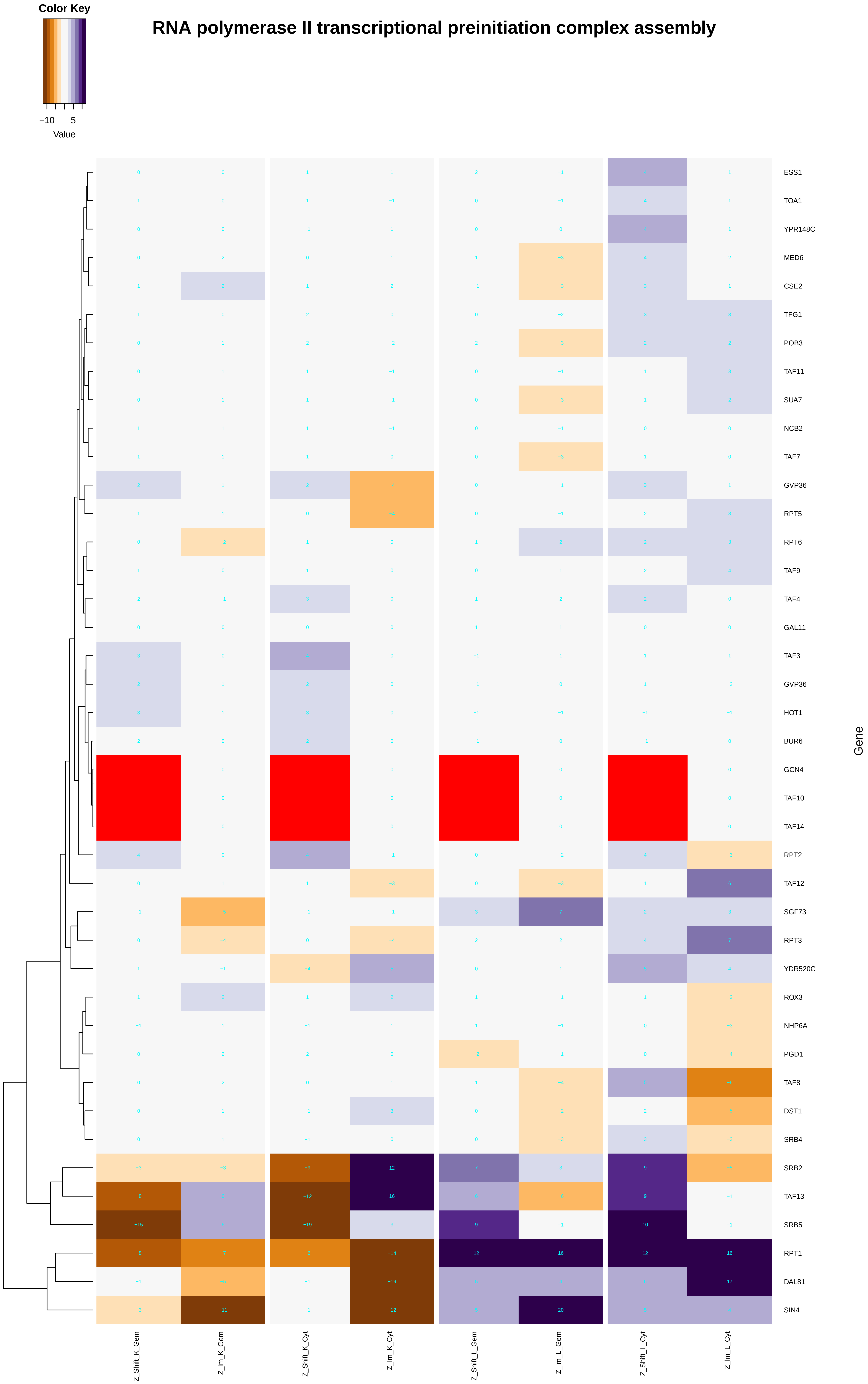

RNA polymerase III transcriptional preinitiation complex assembly

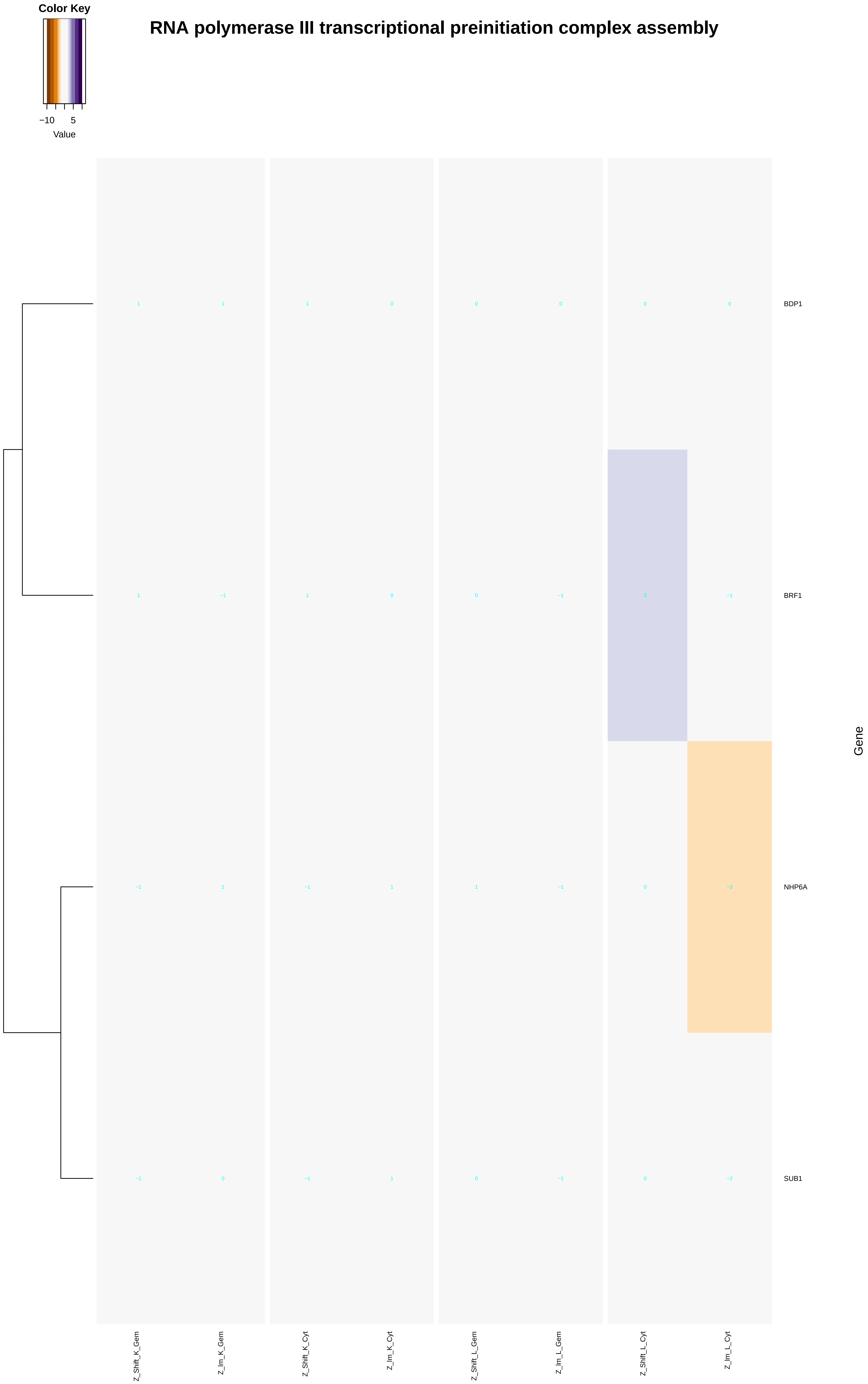

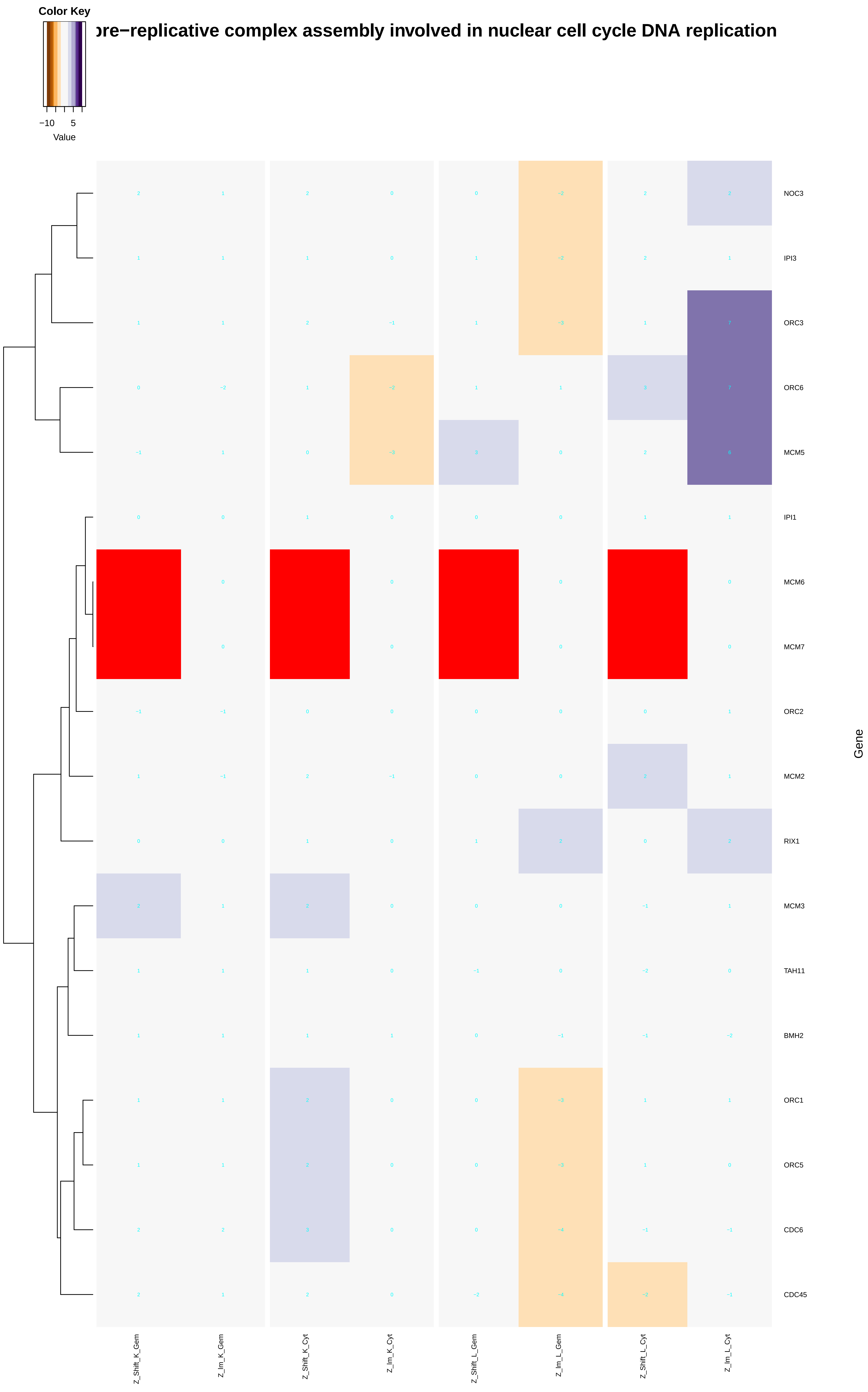

Color Key
−10
5
Value
pre−replicative complex assembly involved in nuclear cell cycle DNA replication
NOC3
IPI3
ORC3
ORC6
MCM5
IPI1
MCM6
MCM7
ORC2
MCM2
RIX1
MCM3
TAH11
BMH2
ORC1
ORC5
CDC6
CDC45
Gene
Z_Shift_K_Gem
Z_lm_K_Gem
Z_Shift_K_Cyt
Z_lm_K_Cyt
Z_Shift_L_Gem
Z_lm_L_Gem
Z_Shift_L_Cyt
Z_lm_L_Cyt
